# *USP19* and *RPL23* as Candidate Prognostic Markers for Advanced-Stage High-Grade Serous Ovarian Carcinoma

**DOI:** 10.3390/cancers13163976

**Published:** 2021-08-06

**Authors:** Haeyoun Kang, Min Chul Choi, Sewha Kim, Ju-Yeon Jeong, Ah-Young Kwon, Tae-Hoen Kim, Gwangil Kim, Won Duk Joo, Hyun Park, Chan Lee, Seung Hun Song, Sang Geun Jung, Sohyun Hwang, Hee Jung An

**Affiliations:** 1 Department of Pathology, CHA Bundang Medical Center, CHA University, Seongnam-si 13496, Gyeonggi-do, Korea; hykang@cha.ac.kr (H.K.); flower0526@hanmail.net (S.K.); kwonahyoung@chamc.co.kr (A.-Y.K.); ice_t69@cha.ac.kr (T.-H.K.); blacknw@cha.ac.kr (G.K.); 2 Center for Cancer Precision Medicine, CHA Bundang Medical Center, CHA University, Seongnam-si 13496, Gyeonggi-do, Korea; oursk79@cha.ac.kr; 3 Comprehensive Gynecologic Cancer Center, CHA Bundang Medical Center, CHA University, Seongnam-si 13496, Gyeonggi-do, Korea; wdjoo@chamc.co.kr (W.D.J.); p06162006@cha.ac.kr (H.P.); chanoncology@chamc.co.kr (C.L.); shsong@chamc.co.kr (S.H.S.); sgoncol@chamc.co.kr (S.G.J.); 4 CHA Advanced Research Institute, CHA Bundang Medical Center, Seongnam-si 13496, Gyeonggi-do, Korea; dufakthd@chamc.co.kr; 5 Department of Biomedical Science, CHA University, Pocheon-si 11160, Gyeonggi-do, Korea

**Keywords:** high-grade serous carcinoma, next-generation sequencing, machine learning, prognostic marker, ovarian cancer

## Abstract

**Simple Summary:**

Although ovarian cancer is one of the leading causes of deaths among female patients with cancer, some patients live longer than others. In order to predict the outcome of patients with ovarian cancer, we investigated the expression levels of all human genes in 51 patients with ovarian cancer and constructed a prediction model based on artificial intelligence. The model identified two genes—*USP19* and *RPL23*—as the most important genes for this prediction. Cancer recurrence occurred more frequently in the patients with lower *USP19* mRNA levels and those with higher *RPL23* mRNA levels. The same pattern was observed in 208 independent patients with ovarian cancer listed in The Cancer Genome Atlas. Therefore, we suggest *USP19* and *RPL23* as candidate biomarkers for predicting the survival of patients with ovarian cancer.

**Abstract:**

Ovarian cancer is one of the leading causes of deaths among patients with gynecological malignancies worldwide. In order to identify prognostic markers for ovarian cancer, we performed RNA-sequencing and analyzed the transcriptome data from 51 patients who received conventional therapies for high-grade serous ovarian carcinoma (HGSC). Patients with early-stage (I or II) HGSC exhibited higher immune gene expression than patients with advanced stage (III or IV) HGSC. In order to predict the prognosis of patients with HGSC, we created machine learning-based models and identified *USP19* and *RPL23* as candidate prognostic markers. Specifically, patients with lower *USP19* mRNA levels and those with higher *RPL23* mRNA levels had worse prognoses. This model was then used to analyze the data of patients with HGSC hosted on The Cancer Genome Atlas; this analysis validated the prognostic abilities of these two genes with respect to patient survival. Taken together, the transcriptome profiles of *USP19* and *RPL23* determined using a machine-learning model could serve as prognostic markers for patients with HGSC receiving conventional therapy.

## 1. Introduction

The precise molecular characterization of tumors from individual patients is important in this era of targeted therapy. Ovarian cancer is a leading cause of deaths among patients with gynecological malignancies, with approximately 240,000 ovarian cancer diagnoses worldwide each year [1,2]. Although new treatment modalities, such as poly(ADP-ribose) polymerase (PARP) inhibitors, have considerably improved the progression-free survival (PFS) of patients with *BRCA*-mutated ovarian cancer, the overall 5 year survival rate of ovarian carcinomas remains below 48% [2,3]. Therefore, it is important to identify new prognostic markers for ovarian cancer, especially for high-grade serous carcinomas (HGSCs) that are usually diagnosed at an advanced stage and are known to be associated with a poor prognosis [2].

With the advent of innovative next-generation sequencing technologies, tumor transcriptome data of patients with HGSC [4] have been used to predict prognosis. Several molecular subtypes of HGSC have been identified [4,5,6,7,8]. For example, The Cancer Genome Atlas (TCGA) [4] identified the following four expression subtypes: (1) immunoreactive subtype, based on chemokine expression; (2) differentiated subtype, based on ovarian tumor marker expression; (3) proliferative subtype, based on proliferative marker expression; and (4) mesenchymal subtype, based on increased stromal components. Subsequently, Chen et al. [5] presented a consensus classifier for these subtypes. However, the clinical relevance of these subtypes is not clear and remains controversial. Therefore, in order to identify new clinically relevant prognostic markers for patients with advanced-stage HGSC treated with conventional therapy, we applied a random forest model to high-throughput RNA-sequencing transcriptome analysis and examined the usefulness of the previous molecular subtypes.

## 2. Materials and Methods

### 2.1. Patients and Specimens

Among patients who had undergone radical hysterectomy with salpingo-oophorectomy and platinum-based chemotherapy for ovarian carcinomas from 2005 to 2014, we investigated samples of 58 patients with HGSC for whom fresh snap-frozen tissue samples were available in the archives of the CHA Bundang Medical Center Biobank (Seongnam, Korea). Among the samples from these 58 patients, we excluded samples from seven patients due the inadequate quality (RNA integrity number, RIN < 6) of RNA after extraction for sequencing. The medical records of the remaining 51 patients were reviewed. HGSC diagnosed based on tumor histology was reviewed by two pathologists (HK and SK). The World Health Organization 2014 classification was used to classify HGSC.

### 2.2. Library Preparation and mRNA Sequencing

RNA was extracted from individual fresh snap-frozen tissue samples by using TRIzol (Invitrogen) according to the manufacturer’s protocol. mRNA was purified and fragmented from total RNA (1 µg) using poly-T oligo-attached magnetic beads. RNA purity was determined using NanoDrop8000 (Thermo Fisher Scientific, Waltham, MA, USA). The total RNA integrity (RNA integrity number ≥ 6.5) was checked using the Agilent 2100 Bioanalyzer. RNA sequencing libraries were prepared using the Illumina TruSeq stranded mRNA Prep kit according to the manufacturer’s instructions. The cleaved RNA fragments primed with random hexamers were reverse transcribed into first strand cDNA. Thereafter, a single ‘A’ base was added to the cDNA fragments, which were subsequently ligated to the adapter. The products were purified and amplified by polymerase chain reaction to create a strand-specific cDNA library. The quality of the amplified libraries was verified by capillary electrophoresis (Bioanalyzer, Agilent, Santa Clara, CA, USA). The libraries were multiplexed and loaded on a flow cell for cluster generation on cBot (Illumina, San Diego, CA, USA). The flow cell was loaded on a HiSeq 2500 sequencing system (Illumina, San Diego, CA, USA). The average sequencing depth was 82 million (2 × 101-bp 41 million paired-end) sequencing reads.

### 2.3. Transcriptome Analysis

All mRNA sequencing steps were conducted according to the guidelines proposed in TCGA mRNA-seq Pipeline for UNC (University of North Carolina) data (https://webshare.bioinf.unc.edu/public/mRNAseq_TCGA/UNC_mRNAseq_summary.pdf (accessed on 21 February 2017)) by using Mapsplice-v2.1.8 and RSEM-1.1.13 [9]. The consensusOV R package was used to identify the molecular subtypes [5]. The edgeR package [10] was used to identify differentially expressed genes (DEGs) in each comparison. Gene set analysis of each DEG was performed using Fisher’s exact test. Gene Ontology [11] and MSigDB 7.0 (molecular signature database) [12] were used as reference pathway datasets. For each gene, the area under the receiver operating characteristic curve (AUC) value, median absolute deviation (MAD), and PFS *p*-value were calculated. For multiple comparisons, we used the Benjamini–Hochberg *p*-value correction procedure. The AUC value ranges between 0 and 1, and a higher value represents better predictability. A greater MAD score represents a higher possibility that the expression of a particular gene can be discriminated among groups.

### 2.4. Identification of Candidate Prognostic Biomarker Genes

A three-step sequential process was employed to identify genes with prognostic significance (based on the (i) MAD score, (ii) AUC score, and (iii) PFS *p*-value). Random forest models using the randomForest R package were generated based on genes that were identified as being significant in the three-step analysis. Random forest represents one of the most successful algorithms for classification and results in accurate and robust predictions based on resampling mechanisms [13]. We set the number of trees to 10,000 and other parameters were set to default values. We created random forest models by increasing the gene number from 2 to 28 (significant genes) and measured the error rates. A higher mean decrease in the Gini score—which indicates how the accuracy of the model would decrease when individual genes are excluded from the model—of the random forest model indicates the prognostic value of individual genes. We selected the top two genes based on the mean decrease in the Gini score for generating and validating the random forest model.

### 2.5. Model Validation

We validated the random forest model using TCGA mRNA expression data (RNAseq v2. RSEM Level 3) for 304 patients with HGSC [4] downloaded from Broad GDAC Firehose (https://gdac.broadinstitute.org (accessed on 28 January 2016)). We selected data of 208 of the 304 patients with stage III or IV disease for whom information regarding PFS was available; PFS data were obtained from the supplementary table from a study on TCGA patients with ovarian cancer [4]. *BRCA1/2* mutation data in the Ovarian Serous Cystadenocarcinoma TCGA dataset [4] were downloaded from cBioPortal [14]. The recurrence probability was calculated by using the random forest model. If the recurrence probability was <80, the patient was categorized as having a good prognosis.

### 2.6. Validation of the Prognostic Value of USP19 and RPL23 Expression

We validated the prognostic value of *USP19* and *RPL23* expression using another HGSC mRNA expression dataset (GSE102094) downloaded from the GEO database. We selected the data of 81 of 85 patients with stage III or IV disease for whom information regarding PFS was available; PFS data were obtained from the supplementary table in Ducie et al. [15]. As GSE102094 included 10 normal samples, we calculated the fold change of each gene based on its normal expression.

### 2.7. Statistical Analysis

All plots (i.e., volcano plots, violin plots, and survival plots) were constructed using the ggplot2 R package [16]. Principal component analysis (PCA) was performed by using the R stats package. AUC values were calculated by using the ROCR R package [17] and the PFS *p*-value was calculated using the log-rank test in the survival R package [18]. Heatmaps were generated by using ComplexHeatmap in R. Survival analysis was performed using the survival [18] and survminer R packages. Statistical comparisons of age and stage between our patients and those whose data were hosted on TCGA were performed in R by using the *t*-test and Fisher’s exact test, respectively. The difference in the expression of candidate prognostic genes based on clinical groups was evaluated using the *t*-test in R. All statistical data were analyzed using R version 3.4.4.

## 3. Results

### 3.1. Clinical Characteristics of Patients with HGSC

We investigated 51 patients with HGSC in our study and 208 TCGA patients. First, we investigated our 51 patients, whose clinical characteristics are listed in Table 1. The average age of the patients was 55 (range: 36–77) years. Most patients (n = 46, 90.2%) possessed advanced stage III or IV.

The patients were divided into the following three groups based on their response to the first cycle of conventional adjuvant chemotherapy (Table 1): no recurrence group—patients with no recurrence at ≥2 years from the end of first-line platinum-based chemotherapy; platinum-sensitive group—patients exhibiting recurrence 6 months after completing chemotherapy; and platinum-resistance group—patients exhibiting recurrence within 6 months from the end of first-line chemotherapy. Approximately 50% of the patients were in the platinum-sensitive group (n = 26, 51.0%). Except for three patients with stage III HGSC for whom recurrence information was not available, patients with advanced stage (III or IV) HGSC (n = 43) were stratified into two groups according to the recurrence status (no recurrence versus recurrence). Patients exhibiting recurrence were found in the platinum-sensitive and platinum-resistant groups.

As most patients were in an advanced stage, in order to eliminate the bias from different stages, we focused on 43 patients with stage III and IV disease and with platinum-sensitivity information (lost to follow up, n = 3). The data of 208 patients with stage III and IV disease from TCGA were utilized as independent validation data. The clinical characteristics of patients (n = 43; enrolled patients; training set) and patients with stage III–IV HGSC (n = 208; TCGA; validation set) are summarized in Table 2. The mean follow-up period for the training set was 45.1 (range 3.6–123.5) months with a median of 40.4 months, and the mean follow-up period of the validation set was 37 (range 0.3–179.2) months, with a median of 31 months. The *BRCA1/2* mutation data of 43 patients from our institute were published in a previous study [19], although this information was missing for one patient. Somatic and germline *BRCA1/2* mutation information was available for 134 patients in the TCGA dataset [4]. No significant differences in age, stage, and PFS were identified between the TCGA patients and our patients; however, the overall survival and the incidence of *BRCA1/2* mutation were significantly different.

### 3.2. Gene Expression Profiles in 51 Patients with HGSC

**Figure 1:**
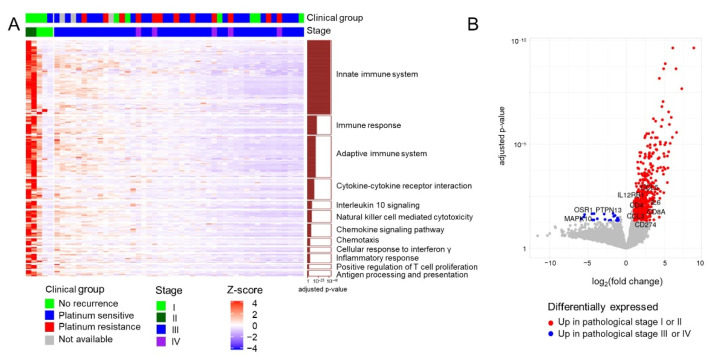
Transcriptome profiles of 51 patients with HGSC. (**A**) Heatmap of upregulated genes in patients with early-stage HGSC. The significant enrichment of individual pathways among the differentially expressed genes is depicted as a bar plot on the right. (**B**) Volcano plot depicting differentially expressed genes between different stage groups (I or II vs. III or IV).

As the responsiveness of the 51 patients to conventional therapy clearly differed according to the stage (Table S1; Fisher’s exact test *p* = 6.06 × 10^−5^), we first identified DEGs between early (I or II, n = 5) and advanced (III or IV, n = 46) stages, revealing 528 upregulated and 22 downregulated genes in patients with early-stage disease based on an adjusted *p*-value of <0.05 and |fold change| of ≥2 (Table S2, Figure 1A,B). The 22 downregulated genes in patients with early-stage disease were not found to be significantly enriched for pathway-related terms. However, the 528 upregulated genes in these patients were found to be enriched in several immune pathway-related terms, such as innate immune response, natural killer cell-mediated cytotoxicity, and chemokine signaling pathway (Figure 1A).

We then investigated the clinical utility of molecular subtypes suggested in previous studies [5,6,7]. The consensus subtypes of Chen et al. [5] could not predict the prognosis of our patients based on the transcriptome data, although these have been claimed to be more reliable subtypes than others [5] (Figure S1A,B). Although the prognosis of the immunoreactive subtype was known to be higher than that of the others, the difference was not significant in terms of overall survival (log-rank test *p* = 0.59) and PFS (log-rank test *p* = 0.58). The expression subtypes proposed by TCGA [4] (Figure S1C,D) also could not predict the prognosis of our patients with HGSC.

The principal component analysis plot did not discriminate the transcriptome profiles among the clinical groups (no recurrence, platinum-sensitive, and platinum-resistance) based on the response to platinum therapy for the 43 advanced-stage patient. Furthermore, no significantly enriched pathway was identified in 100 DEGs among groups (adjusted *p*-value < 0.05 and |fold change| ≥ 2) (Figure S2 and Table S3). Therefore, we stratified our patients with advanced-stage disease into two groups, that is, no recurrence (n = 6) and recurrence (n = 37) for further investigation.

### 3.3. Identification of Candidate Prognostic Biomarker Genes

**Figure 2:**
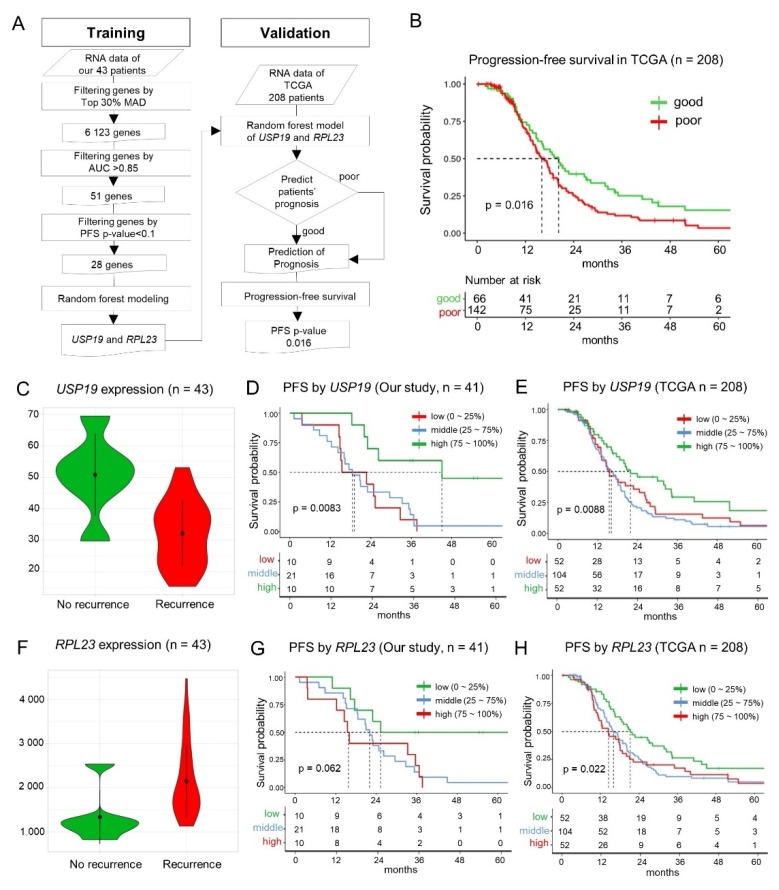
Screening for the prognostic markers and patterns of *USP19* and *RPL23* expression. (**A**) Schematic depicting the workflow used for creating the random forest model and its validation. (**B**) Progression-free survival (PFS) in TCGA HGSC dataset (stage III or IV, n = 208) for validation of the predictive model. (**C**) The expression of *USP19* mRNA in the recurrence and non-recurrence groups (*t*-test *p* = 0.015). (**D**) Graph depicting the correlation between PFS and *USP19* expression in the training set. (**E**) Correlation between PFS and *USP19* expression in the TCGA HGSC validation set. (**F**) The expression of *RPL23* mRNA in the recurrence and no-recurrence groups (*t*-test *p* = 0.020). (**G**) Correlation between PFS and *RPL23* expression in our training set. (**H**) *RPL23*-based evaluation of prognosis in the TCGA HGSC validation set.

As shown in Figure 2A, by using the three-step sequential process for identifying candidate biomarkers in 43 patients with advanced HGSC, we selected 6,123 genes with a MAD score in the top 30%, 51 of which were selected based on a high AUC (>0.85). These 51 genes were then narrowed down to 28 genes based on the PFS *p*-value of <0.1, indicating relevance to prognosis. Although the adjusted *p*-value is a better statistical indicator when making multiple comparisons, none of the 51 genes were found to be significantly related to PFS based on the adjusted *p*-values (<0.1). The details of the 28 candidate biomarker genes are provided in Table S4.

### 3.4. Machine Learning—Based Identification of Key Prognostic Genes

Among the 28 key candidate genes screened as prognostic biomarkers, the random forest model identified *USP19* and *RPL23* as the top two genes based on gene-importance scores for prognosis predictability. As the error rates were very similar among the random forest models with different numbers of genes (median error rate: 11.6%, range: 8.6–13.7%; Figure S3A), we selected the simplest model of the top two genes (Figure S3B) in accordance with the Occam’s razor principle for creating the random forest model. The prediction performance of the random forest model was 11.6% leave-one-out cross-validation error rate, and the sensitivity and specificity were 0.67 and 0.92, respectively.

### 3.5. Validation of the Random Forest Model Employing USP19 and RPL23 Expression

The random forest model that employed the expression of *USP19* and *RPL23* was validated using the PFS data of TCGA patients with HGSC (n = 208) [4] stratified into good prognosis (n = 66) and poor prognosis (n = 142) groups based on probability scores of recurrences. The prognoses of the two groups were significantly different (*p* = 0.016) in terms of PFS (Figure 2B) in accordance with our results.

### 3.6. Prognostic Value of USP19 and RPL23 Expression—Lower USP19 and Higher RPL23 Expression in Patients with Recurrence

In our patients with HGSC, the expression of *USP19* was significantly lower (0.63 times) in the patients with recurrence than the patients without recurrence (*t*-test *p* = 0.015, Figure 2C). We evaluated the PFS of 41 patients among the 43 patients, because the survival data were not available for two patients. The patients were divided into three groups based on the level of *USP19* expression, that is, low (≤25%, 1st quartile), middle (25–75%, 1st–3rd quartiles), and high (>75%, 3rd quartile). The low and middle *USP19* expression groups showed a worse prognosis than the high expression group, and it was statistically significant (n = 43, log-rank, test *p* = 8.3 × 10^−3^, adjusted *p* = 1; Figure 2D). In TCGA patients with HGSC for validation, the low and middle *USP19* expression groups also showed a worse prognosis than the high expression group (n = 208, *p* = 8.8 × 10^−3^; Figure 2E). When we validated it by using another dataset [15] (GSE102094, n = 81), the patients with less than 1.5-fold mRNA expression of the mean of normal samples for *USP19* showed a worse prognosis (*p* = 4.7 × 10^−3^; Figure S4C).

By contrast, the expression of *RPL23* in patients with HGSC recurrence was significantly higher (1.6 times) than the patients without recurrence (*t*-test *p* = 0.020; Figure 2F). Consistently, the high expression group showed a worse prognosis in our patients with HGSC (n = 41, log-rank test *p* = 0.062, adjusted *p* = 1; Figure 2G) and in TCGA patients with HGSC, and it was statistically significant in TCGA data (n = 208, log-rank test *p* = 0.022; Figure 2H). When we validated it by using another dataset (n = 81), the patients with higher mRNA expression than the mean of the normal samples for *RPL23* showed a worse prognosis (*p* = 4.6 × 10^−4^; Figure S4D).

### 3.7. Functional Association between USP19 and DNA Double-Strand Break (DSB) Repair Genes such as BRCA1/2

**Figure 3:**
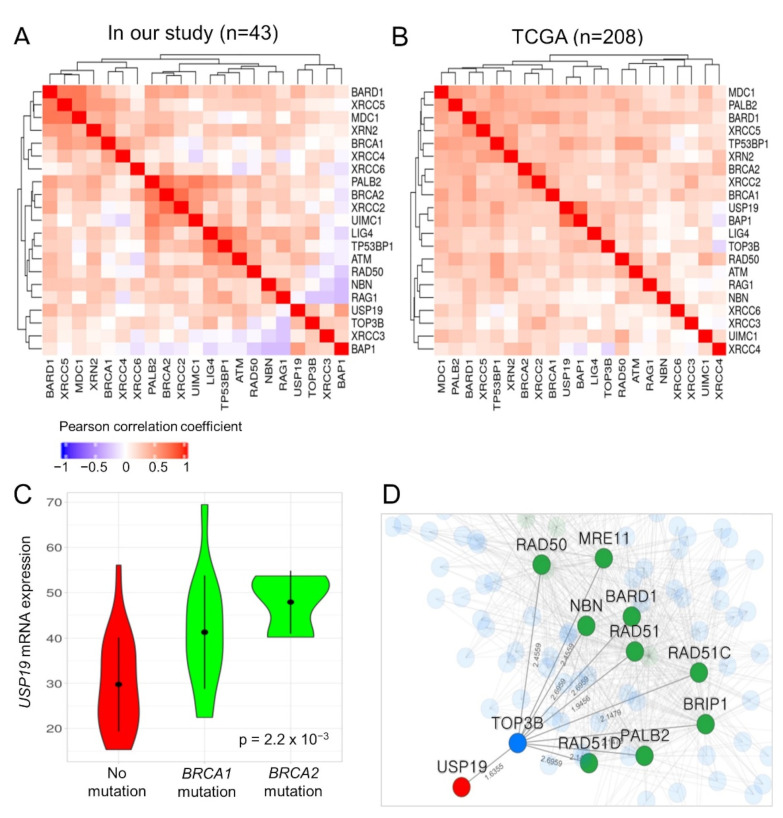
Functional association between *USP19* and DNA double-strand (DSB) repair genes such as *BRCA1/2*. (**A**,**B**) Co-expression pattern among *USP19*, 18 DSB repair genes, and two candidate genes in our patients with HGSC and TCGA datasets, respectively. (**C**) Violin plot depicting *USP19* expression according to the *BRCA1/2* mutation in our patients. (**D**) Sub-network of functional associations among *USP19* and hereditary pathogenic genes responsible for causing breast and ovarian cancer syndrome. Node color represents the characteristics of genes; red: *USP19*, green: DSB repair genes, blue: gene connecting *USP19* to DSB repair genes.

USP19 has been reported to be positively correlated with BRCA1-associated protein 1 (BAP1), a tumor suppressor gene involved in mediating the DSB repair response [20]. In HGSC patients with the *BRCA1/2* mutation, the DSB repair response mechanism is an important target for therapy. Therefore, we investigated the functional association between *USP19* and the DSB repair response. *USP19* was co-expressed with 18 genes involved in the DSB repair response, including *BRCA1/2*, *BAP1* (Pearson correlation coefficient: 0.502, *p* = 6.12 × 10^−4^), *BARD1*, *MDC1*, *RAD50*, *TP53BP1*, and *PALB2* in our patients, as well as in TCGA datasets (Figure 3A,B).

Moreover, our HGSC patients with a mutation in *BRCA1/2* exhibited significantly higher expression of *USP19* mRNA than those without (ANOVA *p*-value: 2.2 × 10^−3^; Figure 3C). The *USP19* mRNA expression was not related to the *BRCA1/2* mutation in the TCGA dataset (ANOVA *p*-value: 0.27). However, in both datasets, the expression of *USP19* mRNA was significantly associated with PFS in patients with HGSC after adjusting for the *BRCA1/2* mutation status in Cox regression analysis (in patients with available *BRCA* mutation and survival data; 42 and 134 patients in our study and TCGA data, *p* = 0.0018 and *p* = 0.017, respectively; Table 3).

Finally, we investigated the functional association between *USP19* and 14 genes (DSB repair genes—*BARD1*, *BRCA1*, *BRCA2*, *BRIP1*, *CHEK2*, *MRE11*, *NBN*, *PALB2*, *PTEN*, *RAD50*, *RAD51*, *RAD51C*, *RAD51D*, and *TP53*) related to hereditary breast and ovarian cancer syndrome in DisGeNet (ID: C0677776) by using the HumanNet v2 functional network (https://www.inetbio.org/humannet/ (accessed on 2 December 2020)), which is a tool for identifying an association between candidate genes and specific diseases using a functional gene network [21]. *USP19* was functionally associated with nine genes, namely *BARD1*, *BRIP1*, *MRE11*, *NBN*, *PALB2*, *RAD50*, *RAD51*, *RAD51C,* and *RAD51D,* through the *TOP3B* gene in HumanNet-XN (Figure 3D). The mRNA expression of *TOP3B* and *XRN2* also positively correlated with that of *USP19* in patients with HGSC (Figure 3A,B).

## 4. Discussion

We sought to identify prognostic biomarkers for patients with HGSC treated with conventional therapy by exploring the gene expression profiles of tumors by RNA-sequencing. In this study, many of the DEGs identified based on disease stage were associated with innate immune response pathways (Figure 1), implying that the innate immune response may play an important role in the prognosis of early stage HGSC. This finding is in agreement with that of a previous study, which demonstrated that a higher number of intratumoral tumor-infiltrating lymphocytes were significantly associated with a favorable prognosis in patients with ovarian cancer [22].

Here, we identified *USP19* and *RPL23* as prognostic biomarkers for advanced-stage ovarian HGSC by using a machine-learning model, and these findings were validated using TCGA data (Figure 2). RPL23 is a ribosome protein (RP), which is considered a nonspecific molecular machine responsible for translating mRNA into proteins. There are approximately 80 RPs in eukaryotic ribosomes, and *RPL23* has been reported to be associated with multidrug resistance and cancer progression. *RPL23* was found to negatively regulate apoptosis via the *RPL23/Miz-1/c-Myc* circuit in a higher-risk myelodysplastic syndrome patient cell line [23], and it could promote multidrug resistance in gastric cancer cells by inhibiting drug-induced apoptosis [24]. According to the human protein atlas (www.proteinatlas.org (accessed on 7 June 2021)), various cancers exhibit a high expression of RPL23, including breast, endometrial, ovarian, and urothelial cancers. In the present study, *RPL23* expression was 1.6-times higher in patients with recurrent HGSC than that in patients without recurrence. Although the survival curve did not show a significant correlation between higher *RPL23* expression and a worse prognosis in our patients with HGSC (n = 41, log-rank test *p* = 0.062, adjusted *p* = 1; Figure 2G), there was a statistically significant correlation between higher *RPL23* expression and poor survival in TCGA patients with HGSC (n = 208, log-rank test *p* = 0.022; Figure 2H). In accordance with our results, Newton et al. reported that *RPL23* was one of the significantly altered genes in the non-responders after first-line chemotherapy (1.5-fold higher than that in responders, *p* = 0.019) when they examined 31 patients with advanced HGSC using the cDNA microarray [25]. Taken together, a higher expression of *RPL23* might induce the recurrence of HGSCs and, consequently, results in worse prognoses in these patients.

Ubiquitin-specific peptidases (USPs) are the main deubiquitinating enzymes (DUBs) that control the activities and levels of proteins regulating many intracellular processes, including cell cycle progression, transcriptional activation, and signal transduction by removing ubiquitin from ubiquitinated substrates [26,27,28]. DUBs are extensively involved in cell cycle regulation, DNA damage repair, and cell growth control [29]. Therefore, DUBs have been recently suggested as potential targets for cancer therapy [30].

Among the approximately 100 DUBs identified in the human genome, 48 were suggested to be the major USPs associated with tumorigenesis. USP19 is one of the putative tumor-suppressive USPs [31]. USP19 physically interacts with and deubiquitinates HDAC1/2 in order to regulate DNA damage repair, chromosomal stability, and tumorigenesis, and USP19 expression is low or depleted in several types of tumors [32]. Therefore, USP19 could be a key factor modulating DNA damage repair by targeting HDAC1/2 K63-linked ubiquitination; cells with USP19 deletion or decreased USP19 expression might exhibit genome instability and even contribute to tumorigenesis [32]. A previous study on the expression of *BAP1* tumor suppressor gene in 1222 patients with TCGA breast cancer data reported that the expression of *BAP1*, which enhances BRCA1-mediated suppression of cell proliferation through BRCA1 stabilization, is highly correlated with *USP19* expression. The study also reported that its expression is lower in dead patients than compared to the survivors of breast cancer and uveal melanoma [20]. Another cancer in which *UPS19* has been reported to be a prognostic marker of is clear cell renal cell carcinoma (ccRCC). Specifically, the major isoform of USP19 (uc003cvz, NM_001199161 also known as isoform 2 of USP19) was significantly downregulated in patients with stage IV ccRCC [32,33], and this was similar to our results in patients with HGSC. Moreover, the downregulation of USP19 promoted tumor growth in a xenograft model. Isoform 1 of USP19 (uc011bch or NM_001199160) has a transmembrane domain for anchoring to the endoplasmic reticulum, whereas isoform 2 contains an EEVD motif [34] and a distinct C-terminal. The functional difference between the USP19 isoforms should be further studied in order to elucidate the molecular mechanism associated with USP19-mediated promotion of cancer pathogenesis.

As for the ovarian cancers, this is the first study to report that *USP19* is a putative prognostic marker, demonstrating that its low expression is significantly related to cancer recurrence and worse prognoses in patients with HGSC receiving conventional therapy.

We also found that patients with the *BRCA1/2* mutation exhibited significantly higher mRNA expression of *USP19* than patients without the *BRCA1/2* mutation in our cohort, but it was not related to the *BRCA1/2* mutation in TCGA datasets. The different results for these two cohorts might be related to the higher (40.5%, 17/42) *BRCA1/2* mutation rate in our patients compared with that in TCGA patient data (9.0%, 12/134) (Table 2).

The HumanNet v2 functional network analysis further revealed that TOP3B and USP19 might physically interact (Figure 3D). TOP3B is a DNA topoisomerase that relaxes the supercoils and alters the topology of DNA. TOP3B is required for preventing the accumulation of excessive R-loops, and its loss results in genome instability and DNA damage [35]. In addition to TOP3B, XRN2 was also predicted to physically interact with USP19, and the loss of XRN2 results in increased DSB formation and genomic instability [36]. Consistent with the findings of the previous study, we found that the mRNA expressions of *TOP3B* and *XRN2*, which regulate genome instability, positively correlated with that of *USP19* in our patients with HGSC.

The DSB repair response is mediated via two major pathways that include the homologous recombination (HR) and non-homologous end joining (NHEJ) [37]. A central function of ubiquitination in DSB repair is to maintain the balance between NHEJ and HR at the S/G2 checkpoint [38]. *USP19* was co-expressed with 18 genes involved in the DSB repair response in our patients as well as in TCGA patients (Figure 3A,B). Therefore, USP19-mediated deubiquitination of key regulators associated with DSB repair or genome instability might be responsible for the worse prognosis of HGSC patients with downregulated *USP19*.

Taken together, USP19 appears to play a key role in tumorigenesis-related processes, such as DNA damage repair and genome instability in many human cancers, including HGSCs and ccRCC. Therefore, we tried to investigate whether RPL23 expression, which is one of our prognostic markers in this study, is also associated with the prognosis of patients with ccRCC as well as HGSC and identified a significant association between RPL23 expression and the prognosis of patients with ccRCC (Figure S5). Considering that HGSC patients with a higher expression of (z-score > 1) RPL23 mRNA exhibited a worse prognosis than other patients in our study and that both ccRCC and HGSC are characterized by high genomic instability, the altered USP19 and RPL23 expressions might be related to genomic instability in cancers.

Some limitations of our study include the small number of our initial samples and the bias between the sample size of groups, which includes early stage (n = 5) vs. late stage (n = 46) and no recurrence (n = 6) and recurrence (n = 37). As most patients with HGSC possess a recurrent disease, it is difficult to obtain many samples from patients with the early stage disease or without recurrence. In order to overcome these limitations, we employed random forest models as a robust model based on resampling methods and validated our findings in 208 TCGA patients with HGSC. However, their molecular and functional mechanisms need to be further investigated.

## 5. Conclusions

We identified *USP19* and *RPL23* as candidate prognostic markers for patients with ovarian HGSC treated conventional therapy by using a machine-learning model, which was validated by using a larger TCGA cohort. Among these two new markers, *USP19* is related to critical known biomarkers of ovarian HGSCs, such as *BRCA1/2*, which is associated with DSB repair mechanisms. Therefore, *USP19* might serve as a new treatment target or a clinical marker when treating HGSC patients with PARP inhibitors.

## Figures and Tables

**Table 1:** Clinical characteristics of 51 patients with HGSC in our study.

Characteristic		Overall (n = 51)	%
Age (Years), Median (Range)		55 (36–77)	
Cancer site			
	Ovary	49	96.0
	Fallopian tube	1	2.0
	Peritoneum	1	2.0
Stage			
	I	3	5.9
	II	2	3.9
	III	41	80.4
	IV	5	9.8
Platinum sensitivity			
	No recurrence	10	19.6
	Recurrence		
	Platinum sensitive	26	51.0
	Platinum resistant	12	23.5
	Lost to follow up	3	5.9

**Table 2:** Clinical characteristics of patients in the training set and validation set for predicting prognosis.

Characteristic		Our Study (Training Set)	TCGA (Validation Set)	*p*-Value
Number of patients		43	208	-
Age (mean years, range)		56 (37–77)	59 (31–87)	0.073
Progression-free survival (median months, 95% confidence interval)		22.8 (18.4–32.8)	17.5 (14.9–19.9)	0.089
Overall survival (median months, 95% confidence interval)		Not reached	45.4 (41.9–53.9)	0.0012
Stage	III	38 (88.4%)	183 (88.0%)	1.0
	IV	5 (11.6%)	25 (12.0%)	
*BRCA1/2* mutation	Germline	14 (32.6%)	1 (0.5%)	9.5 × 10^−6^
	Somatic	3 (7.0%)	9 (4.3%)	
	Germline + somatic	0	2 (1.0%)	
	No mutation	25 (58.1%)	122 (58.7%)	
	Not available	1 (2.3%)	74 (35.6%)	

**Table 3:** Cox regression analysis for investigating the correlation between *USP19* expression and *BRCA1/2* mutation and progression-free survival.

Cohort	Model	Variable	Coefficient	Exp (coef.) = Hazard Ratio	SE (coef.)	*p*-Value
This study (n = 42)	USP19 exp.	USP19	−0.040	0.96	0.014	0.0048 *
	BRCA mutation	BRCA	−0.15	0.86	0.35	0.67
	USP19 exp. + BRCA mutation	USP19 ^#^	−0.056	0.95	0.018	0.0018 *
		BRCA	0.73	2.07	0.45	0.10
TCGA (n = 134)	USP19 exp.	USP19	−0.014	0.99	0.0085	0.091
	BRCA mutation	BRCA	−0.62	0.54	0.35	0.081
	USP19 exp. + BRCA mutation	USP19 ^#^	−0.023	0.98	0.0097	0.017 *
		BRCA	−0.89	0.41	0.38	0.018 *

^#^ USP19 expression after adjusting the *BRCA1/2* mutation status. * *p* < 0.05.

## Data Availability

All expression data are available at GEO Database (https://www.ncbi.nlm.nih.gov/geo/) under accession number GSE165808.

## Supplementary Materials

The following are available online at https://www.mdpi.com/article/10.3390/cancers13163976/s1, Figure S1: Kaplan–Meier plots according to known molecular subtypes, Figure S2: Principal component analysis according to the response groups of platinum therapy, Figure S3: Performance results of random forest models, Figure S4: Kaplan–Meier plots of another independent dataset of patients with HGSC according to *USP19* and *RPL23* fold change based on its normal expression, Figure S5: Kaplan–Meier plots of TCGA patients with renal cell carcinoma according to *RPL23* mRNA expression levels, Table S1: Relationship between clinical stage and response to conventional chemotherapy, Table S2: Differentially expressed 550 genes identified in the comparison of early vs. advanced stages, Table S3: Differentially expressed 100 genes identified in the comparison of response groups of platinum therapy among 43 patients with advanced HGSC, Table S4: Twenty-eight genes associated with prognosis of patients.
