# Supplementary figures and images for: USP19 and RPL23 as Candidate Prognostic Markers for Advanced-Stage High-Grade Serous Ovarian Carcinoma
Source: Cancers (Basel). 2021 Aug 6;13(16):3976. doi: 10.3390/cancers13163976 (PMC8391231; doi:10.3390/cancers13163976)

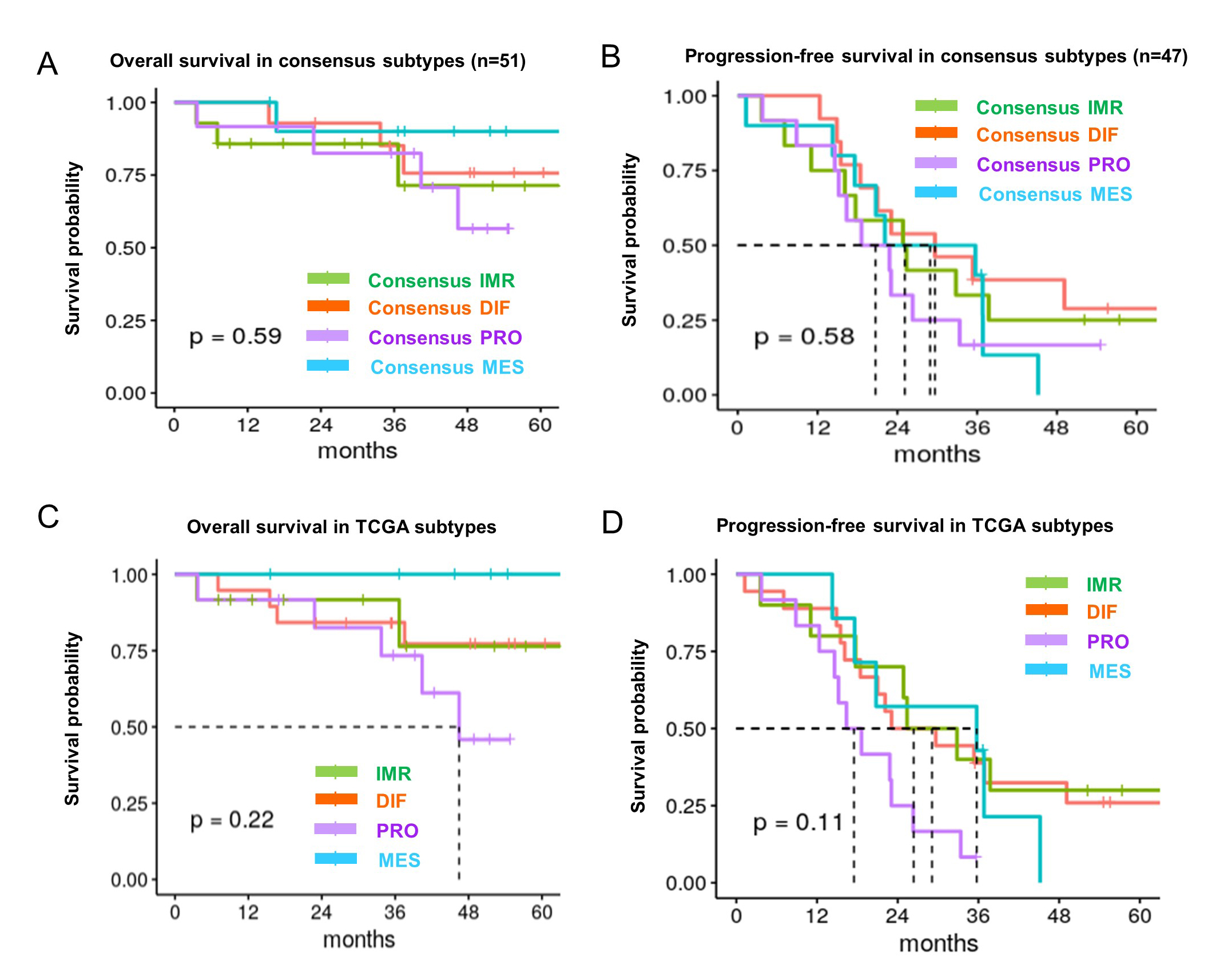

Supplement: Supplementary file 1 [file cancers-13-03976-s001.zip › Supplementary/SFigure1.jpg]

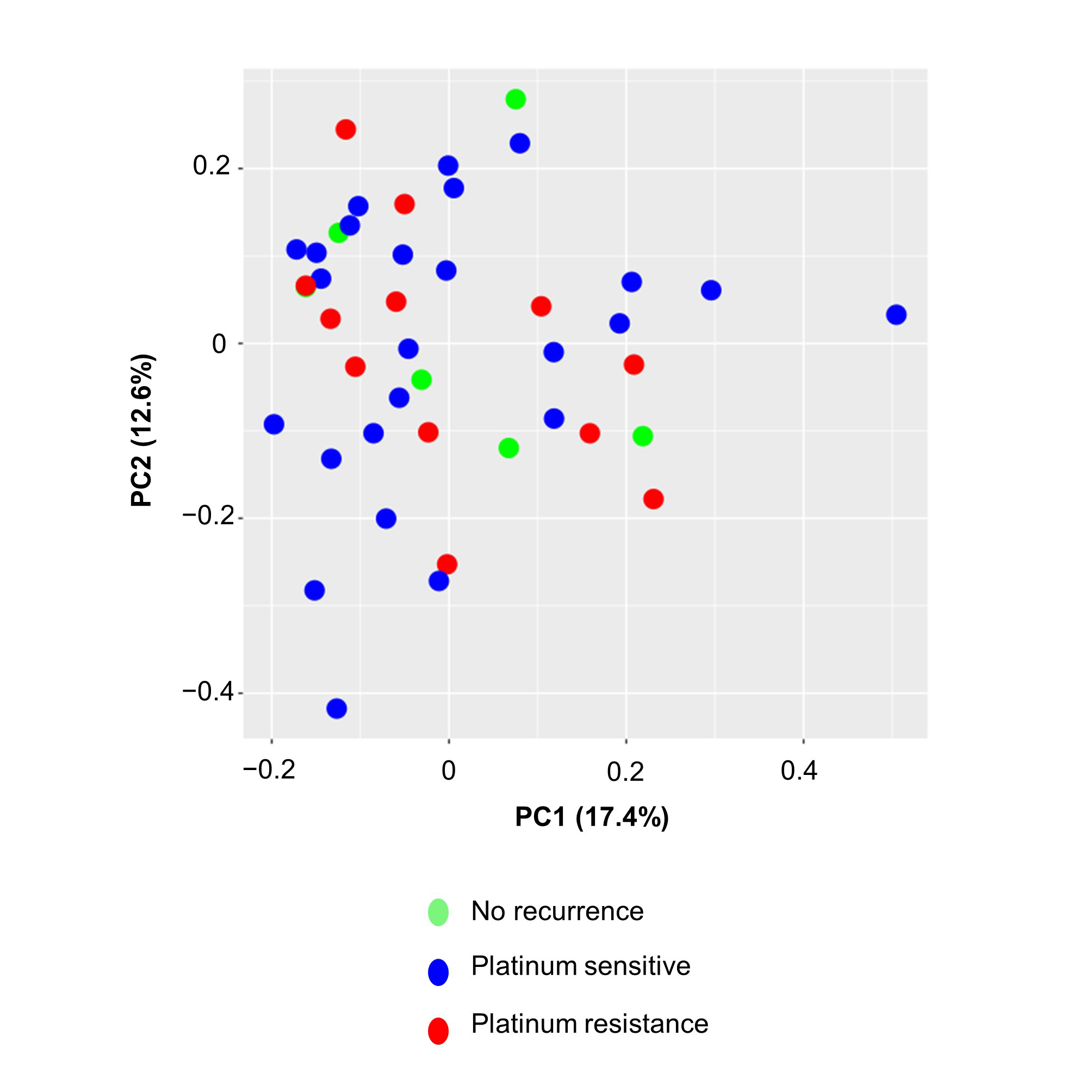

Supplement: Supplementary file 1 [file cancers-13-03976-s001.zip › Supplementary/SFigure2.jpg]

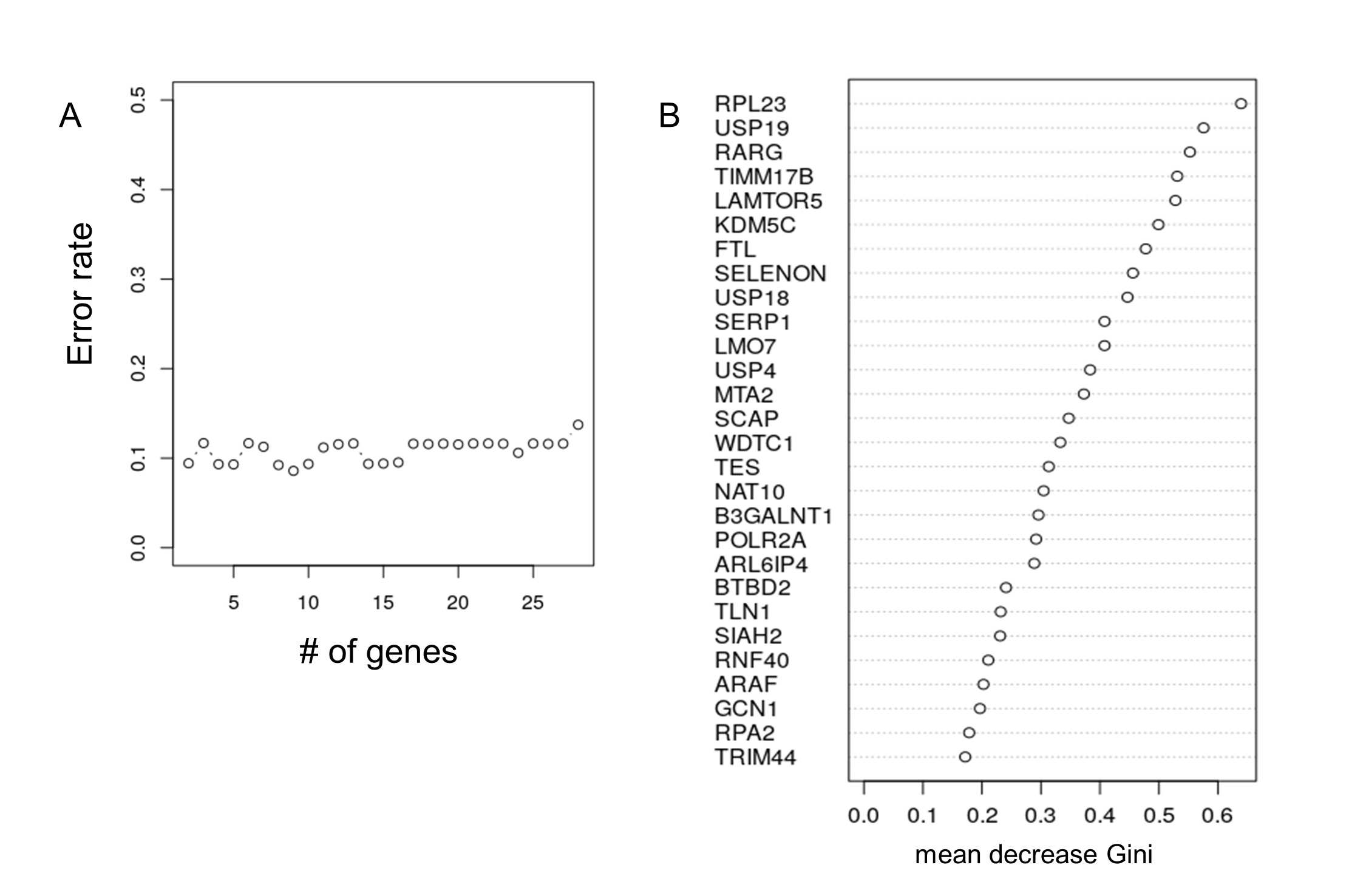

Supplement: Supplementary file 1 [file cancers-13-03976-s001.zip › Supplementary/SFigure3.jpg]

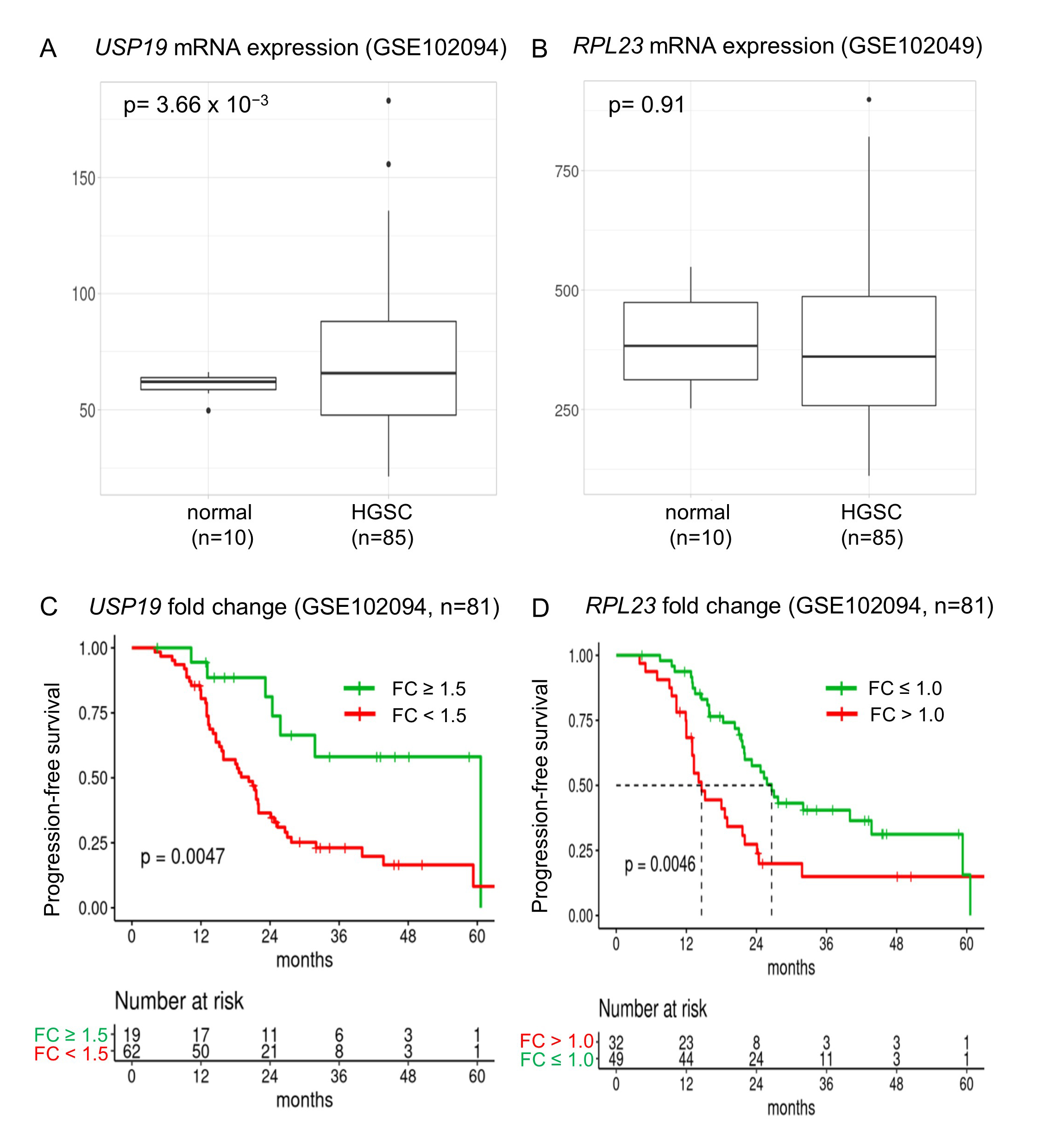

Supplement: Supplementary file 1 [file cancers-13-03976-s001.zip › Supplementary/SFigure4.jpg]

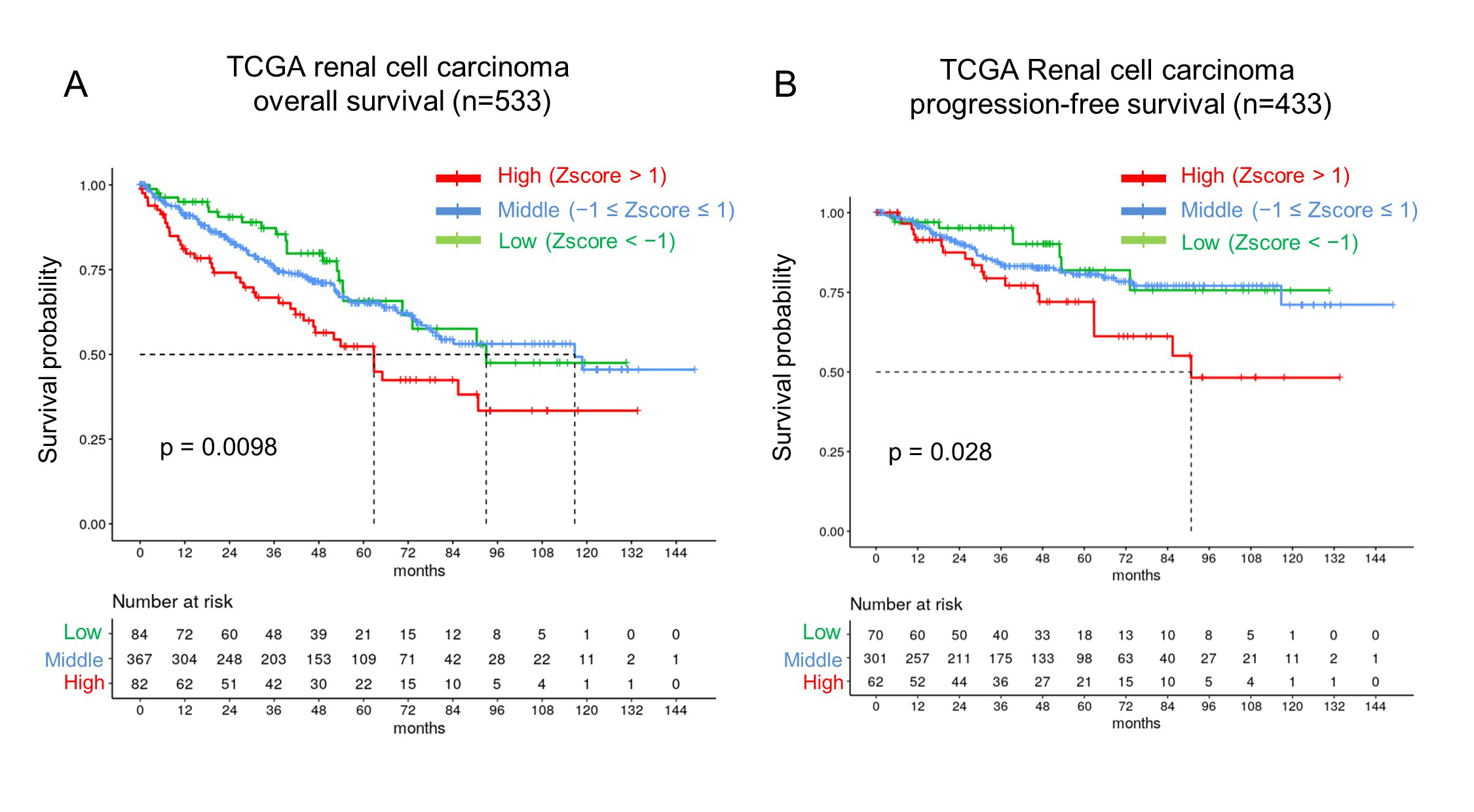

Supplement: Supplementary file 1 [file cancers-13-03976-s001.zip › Supplementary/SFigure5.jpg]
